# Effects of Perceived Benefit on Vitamin D Supplementation Intention: A Theory of Planned Behaviour Perspective

**DOI:** 10.3390/ijerph19041952

**Published:** 2022-02-10

**Authors:** Ying-Hsuan Chen, Shun-Lung Chao, Yen-Wei Chu

**Affiliations:** 1 Ph.D. Program in Medical Biotechnology, National Chung Hsing University, Taichung 402, Taiwan; chen402ys@gmail.com; 2 Taiwan Association of Preventive Health Care, New Taipei 231, Taiwan; p9930@ms25.hinet.net; 3 Institute of Genomics and Bioinformatics, National Chung Hsing University, Taichung 402, Taiwan; 4 Institute of Molecular Biology, National Chung Hsing University, Taichung 402, Taiwan; 5 Agricultural Biotechnology Center, National Chung Hsing University, Taichung 402, Taiwan; 6 Ph.D. Program in Translational Medicine, National Chung Hsing University, Taichung 402, Taiwan; 7 Rong Hsing Research Center for Translational Medicine, Taichung 402, Taiwan

**Keywords:** theory of planned behaviour, vitamin D, perceived benefit, nutrients

## Abstract

There are many factors that affect vitamin D supplementation, including those from the theory of planned behaviour (TPB); however, how the perceived benefit acts in the model remains unknown. In the current study, we tested the efficacy of the TPB and the impacts of the perceived benefit (PBE) in the model. The subjects were 287 customers who purchased vitD from pharmacies in major cities in Taiwan. A structured questionnaire was used to collect the data. *t*-tests, analysis of variance (ANOVA), regression analyses, and path analysis via SPSS and AMOS were used to analyse the data. The original TPB model explained 47.5% of the variance of intention with the three variables of attitude (β = 0.261), perceived behavioural control (β = 0.183), and subjective norms (β = 0.169). The model that incorporated PBE increased the explained variance to 59.7%, and PBE became the strongest predictor (β = 0.310) and a significant mediator linking attitude, subjective norms, perceived control (ANC) with supplementation intention. PBE and attitude were the two most important variables in predicting vitD supplementation intention. We suggest that updated information regarding dietary sources of vitD and its benefits should be included in health- or nutrition-related courses in education programs for the overall health of the nation.

## 1. Introduction

### 1.1. Research Background

Vitamin D (vitD) is an essential nutrient that assists in the absorption of dietary calcium, magnesium, and phosphorus, and is thus vital to the health of human bones, multiple organs, and the immune system [1,2,3,4,5] for adults, mothers, adolescents, and children [6,7]. Recent studies have further indicated that vitD deficiency is associated with risks of numerous musculoskeletal, autoimmune, and cardiovascular diseases [8], as well as cancer, infertility, complications in pregnancy, dementia, and overall mortality (see [2] for details). Although some researchers have used observational or clinical evidence to argue that vitD has weak or no effect on health issues, ranging from health improvement [9] to the incidence and mortality of all diseases and general or site-specific cancers [10,11,12,13,14,15,16,17,18], the importance of vitD as an essential nutrient for human health is confirmed as general wisdom by scientific evidence [2].

Along with the growing literature that has revisited the effectiveness and functions of this nutrient for human health, vitD has been shown to be not only beneficial for skeletal health, as a past study has suggested [1], but also beneficial for the health of human organs. VitD sufficiency is vital and must be maintained through sunshine exposure or dietary supplements [2,3,4,5]. Despite the general public recognizing the importance of vitD for human health, as well as immense associated information exchange in Taiwan, a recent study indicated that significant portions of Taiwan nationals are vitD deficient at a rate of 22.4% of the study population in northern Taiwan. Rates are especially high for those who are female (22.9%), aged between 30–39 years (38.4%), and who hold a graduate degree (31.5%) [19]. The presence of vitD deficiency among these so-called elites in a well-developed area shows that the importance of vitD supplementation has been gravely overlooked, which may expose people and their families to a wide range of vitD deficiency risks. It is thus important for us to examine the factors that affect consumers’ vitD supplementation and to explore whether the perceived benefit (PBE) of vitD plays a role in consumers’ vitD supplementation intention.

Knowledge regarding the benefits of sufficient vitD should be communicated to the nation through health education in any form using updated vitD information to avoid vitD deficiency [20,21]. Core to the success of such health education is to include the factors that affect consumers’ supplementation intention in education or marketing programs [22,23,24]. The current study was conducted to reveal the drivers behind vitD supplementation and to determine whether the perception of vitD benefits may influence the effects of these factors on the outcome. Compared to other theories on health behaviour research, the theory of planned behaviour (TPB) is more explanatory than the others [23]; meanwhile, its simple and efficient framework makes it easier to communicate with participants and researchers. In addition, it is widely used in health promotion studies, such as those on the consumption of vitamins and functional foods, which is in line with the current study [22,23,24,25]. The TPB is thus an optimal theoretical foundation for this research.

In this study, we predicted vitD supplementation intention with the three predictors of attitude, subjective norms, and perceived behaviour. In addition, the results of this study replenish the concept of perceived benefit as a mediator in the TPB model, which helps to further explain the percentage of the variance in vitD supplementation intention.

There are three expected contributions of this study. First, this study may contribute to the literature by providing updated evidence to confirm the efficacy of the TPB in predicting health behaviour and to increase the explanatory capability of the TPB by adding perceived benefits to the model. Secondly, this study may contribute to the healthcare industry by conveying a key message that attitude is the main factor in determining the perceived benefits of vitD and, in turn, the perceived benefits are the pivotal factor affecting the willingness of vitD intake. Third, this study may contribute to health policymakers and practitioners by providing action cues to identify the target markets for effective communication.

### 1.2. Theoretical Background and Hypotheses

In studies explaining healthcare, health promotion, and disease prevention behaviours, the theory of planned behaviour (TPB) has long been widely adopted as a theoretical background for studying diversified kinds of human behaviours [22,23,24,25,26,27]. Prior to the development of the TPB, the theory of reasoned action (TRA) was developed and has shown good levels of efficacy in studying human behaviours [28]. The TPB was then introduced with the additional variable of perceived behavioural control to increase the explaining power of the TRA [29], and it has been widely used to predict intention in human behaviour, including health care, health promotion [25,26,30,31,32], risk avoidance [33], disease prevention [34,35], healthy and sustainable diet [36,37,38], and functional food consumption [39,40]. After close observation, the authors recognized that the TPB was suitable for this study because its concept is well-developed with parsimonious variables that are apt to measure and communicate the study results with academicians and practitioners [23,24,25,26].

The authors of this study attempted to reveal the factors behind vitD supplementation intention based on the widely adopted TPB [22] and to explore the possible effects of PBE in the TPB model.

In the domain of the TPB, there are three independent variables that explain the intention of a targeted behaviour (BI)—attitude (AT), subjective norms (SNs), and perceived behavioural control (PBC). Attitude is the most used determinant among health behaviour theories (HBTs) [30] and is a term in the TPB that refers to a person’s favourable and unfavourable beliefs about a behaviour. Subjective norms are some of the major predictors in HBTs that measure the social influence a person perceives following decision making about a health behaviour [22,30]. Perceived behavioural control appears to be a major advancement in the theory of reasoned action [22], and it refers to a person’s beliefs about control and serves as a proxy for their actual control over a behaviour [22,34]. These three factors then result in the formation of behavioural intention [22]. As previous studies have verified, intention toward the behaviour in question will be affected by the person’s attitude and subjective norms or social influences regarding the behaviour, as well as the perceived behavioural control over the targeted behaviour [25,26,35,37].

In the current study, AT refers to the respondent’s judgment of the vitD supplementation behaviour, SN refers to the effects from the interpersonal network, and PBC denotes the confidence one holds in executing such an intention by considering the difficulties that may be involved with such supplementation behaviour [37], as the model to be shown later as Figure 2 in the result section. These three variables are collectively termed ANC when discussing the concept as a cluster of TPB predictors in this paper. According to the TPB, the three factors of AT, SN, and PBC (ANC) positively affect vitD supplementation. Based on the TPB, ANC positively affects the levels of BI. We thus proposed the following hypothesis:

**H1**. *AT, SN, and PBC positively affect the BI of vitD supplementation intention.*

Since the direction and magnitude of a person’s attitude, perceived social norm pressures, and the perception of control are varied, demographic factors, such as gender, education, and income, have sometimes been included to understand how each construct differs among these categories and to examine how these factors function in a model [25,26,31]. VitD is essential for human health, yet previous studies have shown that vitD deficiency is especially notable in populations with certain demographic characteristics, such as being from northern Taiwan, having higher income, and being well-educated, middle-aged, and female [19]. This means that the determinants of vitD supplementation intention, that is, AT, SN, and PBC, as well as the intention itself, can be different across demographic categories. We thus proposed the following hypothesis:

**H2**. *Levels of AT, SN, PBC, and BI will vary with the respondents’ demographic backgrounds.*

In contrast to adding perceived benefit to the model, past experience was added to the variables explaining behavioural intention (BI) in the TPB around 20 years after its inauguration [22]. We argue that, compared to past experience, perceived benefit is a more logical variable that will extend coverage to those who have not experienced the targeted behaviour. This means that BI may be shaped by perceived benefit rather than by past experience, especially for those who have had no such specific experience. According to theories of rationality, it is logical to predict that human behaviour is the result of rational computation [41,42,43,44]. A person’s actions are motivated by expected benefits. Therefore, it can be inferred that expected benefits will influence an individual’s attitude, social norms, and perceived control over the behaviour that produces the benefits. In other words, perceived benefit is an indispensable factor in the prediction of a benefit-generating behaviour. In the family of HBTs, the health belief model (HBM) is a unique theory that uses the perceived benefit of a healthcare solution as a determinant that combines with susceptibility, severity, and barriers to predict the subject’s intention [30]. Similar to the TPB, the HBM is another widely used theory to predict and explain health behaviour [30,45,46,47,48,49,50], and the perceived benefit of the health behaviour plays an important role in the HBM in predicting intentions [45,46,47,48,49,50]. We borrowed the concept of perceived benefit from the HBM to highlight the importance of benefit perception in predicting health behaviour. Therefore, we inferred in this study that people will engage in proper vitD supplementation if they perceive certain benefits of such behaviour. Thus, we proposed, as part of H4, that the higher the perceived benefit of vitD supplementation, the higher the possibility an individual will engage in vitD supplementation.

People are information processors who encode inputs and compute the outcomes and benefits of each alternative behaviour [51]. The impact of such information on people’s attitudes works through continuous communication with various information sources, such as the media and educational institutes. On the other hand, information that is transmitted to the public will then be accepted by society and form social norms that, in turn, pressure the community members to follow those norms. In addition, people’s confidence in action control rises when information regarding the causes and outcomes of a behaviour is sufficient. Perceived benefit is then formed and increases along with favourable attitudes, social norms, and confidence about control [51]. Humans’ attitudes, norms, perceptions, and confidence toward such behaviour all affect the overall PBE of the behaviour. In the current study, information regarding vitD and its associated benefits for human health has been widely distributed in Taiwan in recent years, which influences people’s attitudes, subjective norms, and behavioural control perception regarding the benefits of vitD. In short, according to the theory of information processing, people’s AT, SN, and PBC regarding vitD supplementation influence their PBE as an outcome. Hence, we proposed H3, as follows:

**H3**. *The higher the levels of AT, SN, and PBC, the higher the level of perceived benefit of vitD supplementation*.

Attitude, subjective norms, and perceived control can affect the BI and PBE, as discussed for H1 and H3, respectively. The PBE will then act as an additional independent variable to influence the BI in the later stage. Since ANC affects PBE, and PBE, in turn, affects BI, the level of PBE has mediating effects on the relationship between the ANC and BI [52]. We thus proposed the following hypothesis:

**H4**. *The higher the perceived benefit of vitD supplementation behaviour, the higher thepossibilityof vitD supplementation intention.PBEmediates the relationships among the variables of AT, SN, PBC, and BI of vitD supplementation intention*.

## 2. Materials and Methods

### 2.1. Data Collection

The research project was approved by the Research Ethics Committee of Jen-Ai Hospital in Taichung, Taiwan with IRB Case No. 109-49 before the study was implemented.

There were 287 subjects who were pharmacy consumers who had consumed vitD products in the metropolitan area of Taiwan in the past six months. The details of the respondent profiles are shown in Table 1. The subjects were volunteers recruited by a printed poster displayed in front of the cashier counter of participating pharmacies around the country. Participants who were younger than 20 years old, pregnant women, aboriginals, prisoners, and others that are not allowed to participate by law or due to legal regulations were excluded. All subjects completed a written informed consent before participating in the investigation with a self-administered questionnaire and were advised that they could terminate the survey at any time. A 10-tablet packet of vitD supplements was provided as a gift for completing the questionnaire.

### 2.2. Research Instrument and Measurement

A structured questionnaire composed of four scales measuring attitude, subjective norms, perceived behavioural control, and behavioural intention was drawn from the TPB questionnaire construction guidelines proposed by an expert in this particular theory [22]. We borrowed the concept of perceived benefit from the HBM [45] and used governmental material to develop a scale as the instrument for perceived benefit.

After the pre-test procedure, we finalized the items on each scale with the expert’s opinions. There are four items to measuring attitude, such as “Regularly taking vitamin D supplements is valuable to body health”, five items measuring subjective norms, such as “My friends in the healthcare industry also take vitamin D supplements to maintain their health”, three items measuring perceived behaviour control, such as “If I wanted, I could take vitamin D each day in the forthcoming month”, and four items measuring behavioural intention, such as “Even if I am out of town for a journey, I will try to take vitamin D supplements each day”. Healthcare experts helped us to identify three groups of benefits from the material that was suggested by the government agency. Three other researchers were invited to validate the scales in the scale development and pre-test stage.

#### Construct Measurement

Attitude is a variable used to measure a subject’s personal judgment of the targeted behaviour; subjective norms is a variable used to measure a subject’s perceived effects from their interpersonal networks, such as colleagues, family members, and healthcare professionals; perceived behavioural control is a variable used to measure the confidence of a subject when facing the difficulties that may be involved with the targeted behaviour [22,23,24,25,26].

PBE was used as a construct to measure how the respondents believed vitD could be helpful in preventing diseases or promoting human health. Perceived benefit items were drawn from the information provided on the National Health Bureau of Taiwan website. The list of perceived benefits ranges from the traditional skeletal health promotion, immunity, and kidney function, reducing the risks of cancers, type 1 diabetes, and allergic reactions, and preventing cardiovascular diseases, dementia, respiratory infections, and more. The list was validated by six pharmaceutical and nutritional experts on a consensus basis.

### 2.3. Data Analysis

Descriptive analysis was used to describe the sample profile; *t*-tests and one-way ANOVA were used to test H2 by examining differences between the average scores for each demographic factor. Multiple regressions were applied to test H1 for the effects of the independent variables AT, SN, and PBC on BI, and to test H3 for the effects of PBE on BI. The significant level was set at *p* ≤ 0.05. Confirmatory factor analysis was applied to examine the reliability and validity of each construct. The bias-corrected percentile of the bootstrapping method was used to determine whether the mediating effect was present by checking the lower and upper bounds of indirect effects. The analytic techniques were applied using the SPSS 22 software package, as well as AMOS 22.0 (IBM Corp, Armonk, NY, USA. Sourced from TriStar, Kaohsiung City, Taiwan). All significance levels were set at *p* ≤ 0.05.

## 3. Results

### 3.1. Participant Profile

There were 287 valid responses gathered for analysis, of which 156 (54.36%) were female, 179 (62.37%) were married, and 179 (62.37%) were aged 20 to 45 years old, as shown in Table 1. Regarding other demographic backgrounds, 79% of subjects were well-educated, with at least a college degree, around 27% of subjects self-reported as retired or acted as housekeepers, and around 37% of respondents earned no more than NTD 35,000 per month (equivalent to USD 1228).

### 3.2. Socio-Demographic Factors

The study used the mean values of each variable to determine whether the values of any particular variables varied with the socio-demographic factors.

The mean value of AT had the highest level in the variable list at 3.94 ± 0.65 on a 5-point scale, as Table 2 shows, followed by PBC (3.91 ± 0.64), BI (3.71 ± 0.69), PBE (3.68 ± 0.69), and SN (3.60 ± 0.67).

The results from the *t*-tests and one-way ANOVA indicate that all variables showed few changes across socio-demographic factors except for gender for AT, SN, and PBE, marital status for SN and BI, income level for PBC, jobs for SN and PBE, and residence area for SN, as shown in Table 2.

Females had higher levels of AT, SN, and PBE than males, and married respondents were higher in SN and BI than those who were single. Regarding age and education level, no significant differences were found. For the income factor, the only significant difference was found in perceived behavioural control, in which the highest income group was much more confident than the lowest group, as shown in Table 2. The significant differences among the categories of some socio-demographic factors in this study are different from those in previous studies [19,25,26,31]. This could be related to the different study samples and contexts.

Job characteristics also mattered. Healthcare professionals were more concerned about the subjective norms than office workers and the jobless. Healthcare professionals also perceived higher benefits of vitD supplementation than the jobless. The statistical analysis also showed that people in southern Taiwan were more concerned about subjective norms than those in the north. The results of the *t*-tests and analyses of variance did not contradict H2 for some demographic factors.

### 3.3. Structural Equation Model

#### 3.3.1. Confirmatory Factor Analysis

A 5-point Likert scale was used to assess the respondents’ perceptions of each construct (items are shown as supplementary material). The instrument has acceptable reliability, with an overall Cronbach’s α = 0.978 (0.950 for construct AT, 0.988 for SN, 0.879 for PBC, 0.978 for PBE, and 0.923 for BI), and all were above the acceptable level of 0.70 for reliability that was suggested by a previous study [53], as shown in Table 3.

**Figure 1:**
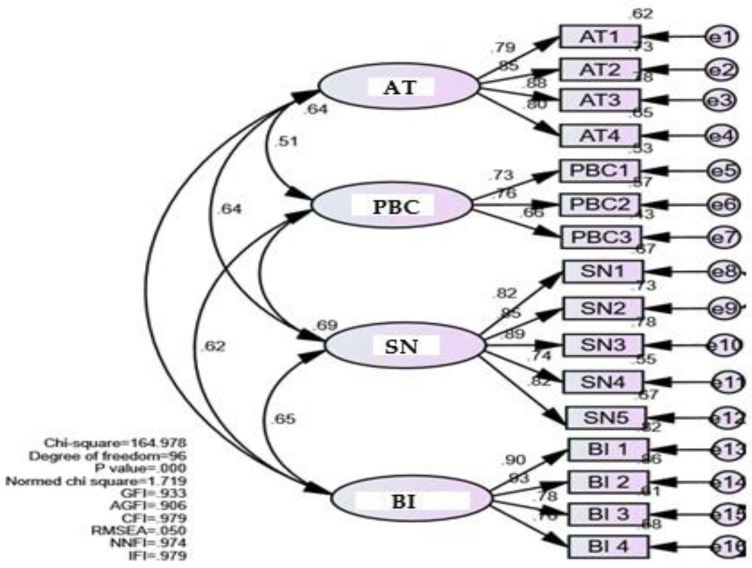
Confirmatory factor analysis; AT = attitude; SN = subjective norms; PBC = perceived behavioural control; BI = supplementation intention; GFI = goodness of fit index; AGFI = adjusted goodness of fit index; CFI = comparative fit index; NNFI = non-normed fit index; IFI = incremental fit index; RMSEA = root mean square error of approximation.

Confirmatory factor analysis (CFA) provided factor loadings, composite reliability, and convergent and discriminant validity of each construct, as shown in Table 3 and Figure 1. The CFA showed good levels of model fit with *X*^2^/*df* = 1.719 < 3.0. Major fit indexes were those larger than 0.9, such as the goodness of fit index (GFI) = 0.933, adjusted goodness of fit index (AGFI) = 0.906, comparative fit index (CFI) = 0.979, non-normed fit index (NNFI) = 0.974, incremental fit index (IFI) = 0.979, and the root mean square error of approximation (RMSEA) = 0.050, as suggested by previous studies [53,54,55,56,57]. Regarding construct validity, Table 3 shows the validity test for each construct. The levels of convergent validity were all at acceptable levels (0.944 for AT, 0.955 for SN, 0.923 for PBC, and 0.950 for BI) of at least 0.5, shown as CR in Table 3. This denotes good levels of convergent validity, as suggested by the literature [57,58,59]. The values of the square root of the average value extract (AVE) of each construct were larger than correlations with other variables, as shown in bold in Table 3. This indicates that all variables have good levels of discriminant validity [57].

#### 3.3.2. Predictors of Vitamin D Supplementation Intention

To test the hypothesized model, path analysis of a structural equation model (SEM) was applied to test the associations among attitude, subjective norms, perceived behavioural control, and supplementation intention, as well as the effects of perceived benefit of vitD supplementation.

**Figure 2:**
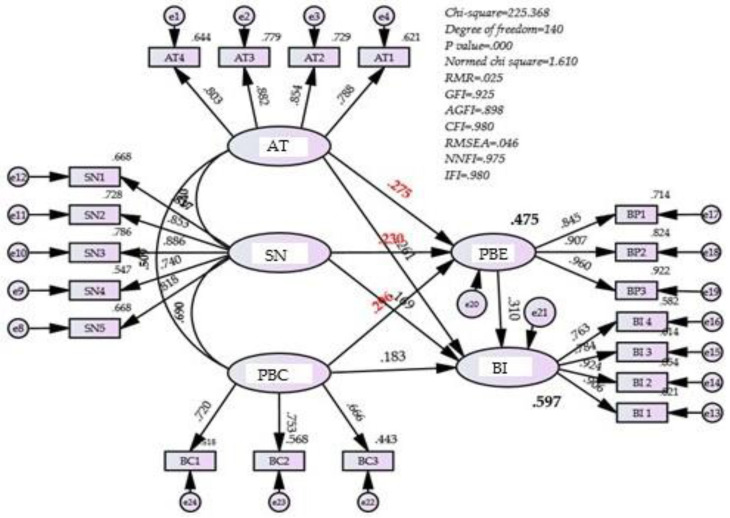
Structural equation model.AT = attitude; SN = subjective norms; PBC = perceived behavioural control; PBE = perceived benefit of vitamin D; BI = supplementation intention; GFI = goodness of fit index; AGFI = adjusted goodness of fit index; CFI = comparative fit index; NNFI = non-normed fit index; IFI = incremental fit index; RMSEA = root mean square error of approximation.

The associations among all variables were significantly correlated, as shown in Table 4 and Figure 2. As per the TPB, vitD supplementation intention was mostly explained by attitudes toward vitD supplementation behaviour (β = 0.261, *p* < 0.001), followed by perceived control (β = 0183, *p* < 0.025) and subjective norms (β = 0.169, *p* = 0.036), which is consistent with previous studies [23,24,25,26,27,37]. On the other hand, attitudes toward vitD perceived benefit (β = 0.310, *p* < 0.001), perceived control (β = 0.275, *p* < 0.001), and subjective norms (β = 0.296, *p* < 0.001) had significantly positive impacts on the perceived benefit of vitD, with 47.5% of the variance explained (R^2^ = 0.475), and perceived behavioural control was the factor that explained most of the variance in perceived benefit.

Regarding the goodness of fit indices for the levels of fitness of the data and the model, the results of the structural equation model show associations with each independent and dependent variable with good levels of model fit, such as *X*²/*df* = 1.610, RMR = 0.025; GFI = 0.925, AGFI = 0.898; CFI = 0.980, RMSEA = 0.046; NFI = 0.975, IFI = 0.980 [53,54,55,56].

#### 3.3.3. Perceived Benefit Mediates the Effects of Vitamin D Supplementation Intention

We further explored the possible mediating effects on the relationship between ANC and BI. The current study hypothesized that the respondents’ perceived benefit of vitD may mediate the links between ANC and vitD supplementation intention.

To test the mediating effects, we applied a bootstrapping method that has been generally accepted as a better method for indirect/mediation evaluation [60,61,62]. Bootstrapping mediation analysis provides confidential intervals to examine the indirect effects. The preferable bootstrapping method is bias-corrected bootstrapping [62]. The bootstrapping analysis with bias-corrected percentile methods (BCPM) showed the values of the lower and upper bounds of the 95% confidence intervals. The standardized indirect effects that PBE brought to the model are shown in the last column of Table 4 as the lower and upper bounds for each of the independent variables. These are 0.030 and 0.176 for AT, 0.015 and 0.156 for SN, and 0.037 and 0.179 for PBC. As there were no zeroes in each interval pair, we are confident that the indirect effect is not zero [52,61,62]. The mediation effect of PBE was thus confirmed for the links between BI and AT, BI and SN, and BI and PBC. H4 of the current study was thus supported [52], as shown in Table 5. This means that the respondents’ PBE transmitted the effects of AT, SN, and PBC to vitD supplementation intention, as shown in Figure 2. After adding PBE as a mediator, the coefficient between PBE and BI was 0.310, and the variance explained increased from 47.5% to a higher level of 59.7% (R^2^ = 0.597), as shown in Figure 2.

## 4. Discussion

This study confirmed that attitude, subjective norms, and perceived behavioural control positively affect vitD supplementation intention with good levels of variance explained (R^2^ = 0.475), as we expected in Hypothesis 1. The results are consistent with those of previous studies [23,24,25,26,30]. Attitude is the strongest predictor and is followed by perceived behavioural control and social norms. Social norms or social influences from similar behaviours performed by peers and family members, as suggested by the social learning theory [25,63], affect a person’s behaviour via learning by imitation and attempts to conform to the group. Social norms have been criticized as a poor predictor in the TPB because respondents in a study may be affected by different types of norms [64,65]. The current study investigated the respondents’ perceived norms via varied sources to include social identity [64] and gain accurate measurements. As a result, social norms appear to have the weakest effect on intention [30].

The predictors and intention vary significantly with the socio-demographic factors of gender and occupation only, and Hypothesis 2 is partially supported. This somewhat deviates from previous studies in which the results have varied by age [19,26] and education levels [19,31]. As for Hypothesis 3, the test result shows that perceived benefit had significant impacts on vitD supplementation intention, and Hypothesis 3 is supported. In addition, the current study confirms the mediating effects of perceived benefit on the associations between AT, SN, PBC, and BI, as predicted in Hypothesis 4. Perceived benefit also increased the percentage of variance explained for vitD supplementation intention.

### 4.1. The Role of Socio-Demographic Factors

Socio-demographic factors shape human behaviour through various mechanisms and thus play important roles in the research of health behaviour [66,67]. In the current study, we hypothesized that attitudes, subjective norms, perceived behavioural control, perceived benefit, and intention of vitamin D supplementation would vary across all socio-demographic factors, yet the results only show partial support for this hypothesis.

As far as gender is concerned, women appear to have more favourable attitudes, subjective norms, and perceived benefit of VitD supplementation, yet are not higher in behavioural intention than men. According to previous studies, women (especially those who are middle-aged) are inclined to ignore sources of vitD and its intake and are exposed to the risks of vitD deficiency [18,19,31]. Given that middle-aged women are usually primary health care decision makers in families [68,69] and are thus in a key position to make decisions about their family members’ disease prevention, health promotion, medical treatment, and so on, middle-aged women’s impact on the health of family members is apparently superior to that of other members [54]. Women’s low intention for vitD supplementation shown in this study is in line with previous studies on vitD deficiency in Hong Kong and Taiwan [19,70], which may suggest that a gap between knowledge and behaviour exists in these family health decision makers [70].

The popularity of higher education in Taiwan is related to the fact that families in Taiwan have always placed great importance on their descendants’ education. The distribution of education levels in this study shows that more than 87% of the subjects have received at least a college education, which reflects higher education numbers in Taiwan. However, a person’s level of health literacy and knowledge is not necessarily consistent with their level of education [70,71]. In other words, higher education may not simultaneously increase appreciation for vitD and knowledge about its benefits and the necessity of vitD supplementation, as previous studies have also revealed that there is no association between knowledge and education [70,71,72].

It is worth noting that people in the northern part of Taiwan had the highest proportion of vitD deficiency [19]. The area covers the state capital, a world-class science and technology park known as Taiwan’s Silicon Valley, with abundant technology and health care resources. Compared to the south and central parts of Taiwan, people in northern Taiwan share almost half of the population (46.7%), are younger on average, and have higher income levels, with much better accessibility to job and education opportunities, as well as foreign cultures [73]. In other words, this is an elite area that features the highest levels of technology, education, and culture in Taiwan. Ironically, this area is also the area with the highest rate of vitD deficiency. Despite the fact that the demographic profile of northern Taiwan differs from other areas of the country, no significant differences were found for each variable except regarding subjective norms.

Given that highly educated women in the northern metropolitan area of Taiwan have the highest proportion of vitD deficiency (as noted in a past survey [19]), the results of the current study further confirm that this particular group is not significantly different from other populations regarding the power of predictors of vitD supplementation intention. This may suggest that vitD deficiency prevails over a wide range of ages, education levels, income levels, and residence areas, though some differences may exist. The population exposed to the health risk of vitD deficiency may far exceed what has previously been estimated.

It is worth noting that as the results of the current study indicate, married women have a greater willingness to supplement vitD and are more affected by the social atmosphere. This implies that healthcare authorities should target married women as the main agents to encourage family members to maintain adequate vitD, as should other healthcare professionals for any campaigns promoting vitD supplementation [70,71,72,74]. Updated information about how vitD supplementation is beneficial to the health of family members should be carefully encoded as the theme of the message. Since married women are greatly affected by social norms, messages of this kind should be delivered through interpersonal networks. Promoting vitD supplementation in these ways could ensure that twice as much is accomplished with half the effort.

### 4.2. The Role of Perceived Behavioural Control

The major difference from the theory of reasoned action (TRA) is that the TPB includes perceived behavioural control as an additional determinant of intentions [29]. Even though the TRA had been highly successful in health-related studies, there are plenty of studies suggesting that PBC significantly increases the explained variance of behaviour [28]. PBC remains a significant predictor in explaining vitD supplementation intention, yet was not a major predictor in this study, which deviates from previous studies [26,34]. Adding perceived behavioural control appeared to be a major advancement of the TPB from the TRA [25,26,75,76], but this was not true in our study.

The test results indicate that the respondents’ AT had a higher average score than the other independent variables. This reveals that the respondents generally possessed positive attitudes toward vitD supplementation behaviour. Even though the importance and health benefits of vitD have been widely communicated to the public in recent years, the PBE’s low average score may indicate that consumer knowledge about vitD has not simultaneously been updated. It is worth noting that 23% of the respondents were healthcare professionals and 78% were well-educated with at least a college education. SNs play a little role in affecting supplementation behaviour, which suggests that consumers’ vitD supplementation intention is least driven by their interpersonal networks. The fact that PBC was the second-highest score can be interpreted as suggesting that these respondents are confident in their subjective knowledge and believe they have no problems with such decision making; this is especially true for healthcare professionals.

Compared to residents in central and northern Taiwan, people in the south are more subjective-norm-oriented or are more easily affected by the social atmosphere. Health information through opinion leaders can be more effective in enhancing vitD supplementation intention in this area. This implies that the choice of channel for health information delivery needs special consideration.

The effects of ANC on BI vary from one to another in previous studies, yet most of them affirmed that PBC is the main predictor [25,26]. In contrast, some of the literature has suggested that the effects of PBC on BI is not apparent [77] or that past experience should be added as an additional variable to predict the targeted behaviour [22,23,24]. The current study shows that PBC remains a reliable yet not major predictor of vitD supplementation intention. This may stem from the fact that the updated information regarding the effectiveness of vitD has not been commonly accepted by the public.

As far as subjective norms are concerned, social learning theorists have suggested that humans learn through observing and imitating adults or teachers [78]. In the socialisation process, people also learn how to react to health problems through observing and imitating healthy people and healthcare professionals [22,45]. This is how subjective norms may drive vitD supplementation intention.

### 4.3. Perceived Benefit as a Mediator

Previous studies have included additional predictor variables, such as past experience [26] or self-efficacy from social cognition theory [78,79,80], in an attempt to enhance the predicting capability of the TPB. The current study borrowed the concept of perceived benefit from the HBM as a mediator. To our knowledge, this is the first study that used perceived benefit as a predictor and consequently provides a better explanation. The PBE of vitD supplementation has been shown to mediate the relationship between ANC and intention. Although the TPB has been confirmed to be applicable in explaining vitD supplementation intention, the current study further suggests that PBE is an important factor that mediates the relationship between ANC and BI. ANC is vital to BI; however, PBE plays a vital role as a mediator in catalysing the effects of ANC on vitD supplementation intention.

The TPB is one of the most popular theories in behaviour studies [20,21,22,23,24]. There have been many attempts to expand the explaining power of conventional theories, which have been highly successful in explaining behaviour. For example, the integrated behaviour model (IBM) adds the concept of self-efficacy (SE) [80] to the TPB to create a new theory [61], and other studies have attempted to add new constructs, such as knowledge, health literacy [66,81], past experience [22,25,26], and more (see [26] for details). The current study included the idea of the perceived benefit of HBM to explore how the new construct may affect the TPB’s core variables (AT, SN, and PBC). The results of this study show that PBE can be used in the TPB as a reliable variable that predicts health-related behaviour.

Vitamin D has long been accepted as an essential nutrient for skeletal health. Thanks to the advancement of research technology, modern studies have shown that vitD is beneficial to a much wider range of human health, including strengthening all kinds of organs [1,2,3,4,5,6,7,8]. Although knowledge regarding the usefulness of vitD has been significantly extended, the general public has not yet fully embraced such updated knowledge. As a result, levels of the perceived benefit of vitD vary across different demographic factors, as does the associated vitD supplementation behaviour.

Despite some arguing that the functions of vitD in human health are exaggerated [9,10,11,12,13,14,15,16,17,18], the literature generally agrees that vitD is an essential nutrient at optimal levels, and vitD deficiency may expose humans to all kinds of health risks [1]. This implies that including updated information about vitD in health education programs is important. In particular, information regarding the importance of vitD for both skeletal and non-skeletal health, dietary sources of vitD, and the prevalence of vitD deficiency. Educational programs of this kind should be provided in health- and nutrition-related courses, such as health education, and as a part of general physical education at all school levels, as well as part of patient education in both outpatient and inpatient sections of a healthcare institute. To be effective or successful in such vitD nutrition- or health-related education, understanding the influential factors from the current study will be important when designing these programs.

### 4.4. Limitations of the Study and Future Research Directions

The current study has some limitations. First, the PBE items in this study were derived from the publications of the National Health Bureau of Taiwan. This means that such items may be culture- or area-specific and may not apply to other areas that are different in terms of country size, cultural background, lifestyle, and geographic distribution, among other factors.

Subjects included in this study were well-educated, which conforms to the current educational levels in Taiwan, and the test results indicate no significant differences across all categories of education and age; however, this may not apply to other countries with different educational and age distributions. Further, one-fourth of the subjects were healthcare professionals, and thus, careful interpretation of the results is suggested.

Although the concept of PBE was borrowed from the HBM to capture the respondent’s perceived benefit of vitD, this is not a solution for any specific health problems. Given that the PBE in HBM was created as a solution to a specific health problem, we are not able to argue that the PBE of vitD supplementation is identical to that in the HBM. Therefore, a careful interpretation may be necessary.

As far as knowledge about vitD is concerned, past studies have proposed and tested the idea that both objective and subjective knowledge significantly shape an individual’s attitudes and associated behaviour, such as regarding organic product consumption [81], but the present study did not include knowledge as a factor in the model. Instead, the concept of vitD benefit perception included in the present study can be viewed as a representation of subjective knowledge or user experience. Although the items on vitD benefits were evaluated by professionals who agreed upon them as objective measures, the current study did not compute or record the correctness of each answer as a measure of objective knowledge. This means that the effects of objective knowledge were not apparent in this study.

As a widely accepted theoretical model for explaining health behaviour, the TPB has exhibited tremendous achievements in a wide variety of research contexts. However, as past studies have mentioned, some limitations originate in using the TPB as a theoretical background, which are addressed here. The TPB was found to be context-specific [82]; hence, the generalizability of this study is limited to similar contexts. Next, intention as the dependent variable in the present model may not perform as an actual vitD supplementation behaviour in the future. Hence, the results of the tests of intention are not directly inferred to represent actual behaviour. Third, we included the perceived benefit of the positive outcomes of vitD supplementation in the current model but did not consider the effects of the negative outcomes of not performing a behaviour on the targeted behaviour. Hence, the test results should be interpreted with care.

We are aware of Reichenbach’s principle [83] on the direction of time; thus, the terms “affect”, “effects”, “predictor”, and “prediction” used in this article do not claim to determine the causality of independent and dependent variables.

Similar to the fact that perceived benefit mediates the relationship between predictors and intention of vitD supplementation, knowledge and health (and/or nutrition) literacy may also have significant effects on health behaviour. Knowledge and health literacy are important factors that may affect humans’ health behaviour and are not necessarily correlated with socio-demographic factors, such as education or income levels [67,78]. It will be interesting to understand what and how the knowledge of vitD and its supplementation can affect consumption behaviour. It will be also interesting to explore the current levels of people’s health and nutrition literacy, and how such levels affect vitD supplementation behaviour.

Knowledge of vitD and awareness about the importance of vitD sufficiency for human health is one thing, whereas the actual behaviour of vitD supplementation is another. This gap between knowledge and behaviour is apparent in many Asian countries [19,63]. Avoiding sunlight to gain white and tender skin is one reason for this gap, as many studies have indicated [65], yet we believe there are more possible reasons for this fact. Are there any existing barriers that help to create such a gap, and how can the knowledge be strengthened to overcome such barriers? Since knowledge is a complicated issue that may include how the knowledge is gained, accumulated, explored, exploited, and transferred, especially in the field of the nutritional function of vitD in human health, it will be an important topic for future research.

Compared to other non-healthcare occupations, healthcare professionals appear to perceive higher social pressures as well as higher perceived benefits. Given that healthcare professionals are part of the national healthcare system and are always obliged to deliver healthcare knowledge or information to patients and the general public, it will be interesting to explore whether individuals with different types of healthcare jobs and at different institutes perceive similar benefits of vitD. Results from studies of this kind will be useful for all levels of school as well as healthcare institutes when designing health education programs specific to enhancing people’s health or nutrition knowledge.

Taken that the married women of middle age are the major healthcare decision makers in a family, what the factors are and how these factors may affect their decision-making process will be worthy for research.

Although the values of various demographic variables are already known, the impacts of the differences in these two regions remain unknown. Regarding the possible impact of demographic differences between the northern and southern regions of Taiwan, it may be worthwhile to further investigate them in detail.

## 5. Conclusions

Research has confirmed that vitD benefits human health far beyond the skeleton system. VitD deficiency has become a remarkable phenomenon in Taiwan. Proper supplementation of vitD is an effective alternative to dietary intake for this particular problem. The current study borrowed the concept of perceived benefit from the HBM to incorporate the three predictors of the TPB to explain vitD supplementation intention. Perceived benefit, as the test results indicate, is the major contributor to an individual’s vitD supplementation intention, and thus deserves to be strengthened by specifically designed education in both schools and healthcare institutes. Attitude is the strongest predictor among the TPB predictors and is followed by perceived behavioural control and subjective norms. Unlike many previous studies that have claimed that perceived behavioural control is the most significant factor in affecting one’s intention, it is third in contributing effects to intention in this study. This may suggest that the respondents of this study were overconfident about their subjective knowledge, and thus ignored the need for vitD supplementation. Social norms contribute the least to intention. An individual may be influenced by their peers and friends to consume vitD supplements, but the effect is limited. This means that social influence does not matter as much as the other behaviours.

Attitude, social norms, and perceived behavioural control were also valid predictors of the levels of perceived benefit of vitD supplementation intention, among which perceived behavioural control was the strongest predictor; again, social norms remained as the weakest. Perceived benefit mediated the associations among attitude, subjective norms, perceived behavioural control, and vitD supplementation intention, and boosted the percentage of variance explained by the TPB from 45.7% to 59.7%.

This study indicates that to effectively improve an individual’s vitD supplementation intention to a satisfactory level, perceived benefit and attitude are the two most important factors, and they are closely related to an individual’s level of knowledge about vitD benefits. This implies that information regarding vitD, specifically dietary sources and the benefits of vitD, should be regularly updated and distributed throughout nutrition- and health-related courses such as health education or physical education at all levels of schooling as well as throughout patient education programs at healthcare institutes to enhance the nation’s vitD knowledge and effective vitD intake. Since numerous efforts via commercial media in the private sector have not resulted in satisfactory outcomes regarding vitD levels, governmental support will be vital for the success of a program of this kind in building healthy vitD levels in the population.

## Figures and Tables

**Table 1:** Participants’ profiles.

Factors	Categories	*n	%
Sex	Female	156	54.36
	Male	131	45.64
Marital status	Single	108	37.63
	Married	179	62.37
Age	20–30	54	18.82
	31–35	42	14.63
	36–40	44	15.33
	41–45	39	13.59
	46–50	32	11.15
	51–55	35	12.20
	56 and above	41	14.29
Education	High School or lower	66	23.00
	College	166	57.84
	Post-graduate	55	19.16
Income	USD 1228 or lower	105	36.59
	USD 1229–1754	75	26.13
	USD 1789–2280	48	16.72
	USD 2315 and above	59	20.56
Jobs	Office jobs	46	16.03
	Healthcare	67	23.34
	Commerce	56	19.51
	Traditional	40	13.94
	None	78	27.18
Area	North	171	59.58
	Central	30	10.45
	South	86	29.97

*n is sample size: 287.

**Table 2:** Construct variance varied in gender, marriage, income, job, and area.

Var	M	SD	Gender ^a^	Marital ^b^	Age	Edu	Income ^c^	Job ^d^	Area ^e^
AT	3.94	0.65	F > M (*p* = 0.014)	n. s.	n. s.	n. s.	n. s.	n. s.	n. s.
SN	3.60	0.67	F > M (*p* = 0.039)	M > S (*p* = 0.025)	n. s.	n. s.	n. s.	HC > O, N (*p* < 0.001)	S > N (*p* = 0.012)
PBC	3.91	0.64	n. s.	n. s.	n. s.	n. s.	H > L (*p* = 0.011)	n. s.	n. s.
PBE	3.68	0.69	F > M (*p* = 0.009)	n. s.	n. s.	n. s.	n. s.	HC > N (*p* = 0.034)	n. s.
BI	3.71	0.69	n. s.	M > S (*p* = 0.005)	n. s.	n. s.	n. s.	n. s.	n. s.

Only significantly different values are shown; M = mean; SD = standard deviation; n. s. = non-significant; AT = attitude; SN = subjective norms; PBC = perceived behavioural control; PBE = perceived benefit of vitamin D; BI = supplementation intention; ^a^ F = female; M = male; ^b^ M = married; S = single; ^c^ H = monthly income at least NTD 66K; L = monthly income no more than NTD 35K; ^d^ HC = healthcare; O = office worker; N = none; ^e^ N = north; C = central; S = south.

**Table 3:** Means, standard deviations, reliability, and validity of the study variables.

Factors	M	SD	Cronbach’s α	CR ^a^	AT	SN	PBC	BI
AT	3.94	0.65	0.950	0.900	**0.833**	0.639 **	0.507 **	0.638 **
SN	3.60	0.67	0.955	0.913	0.639 **	**0.824**	0.687 **	0.643 **
PBC	3.91	0.64	0.879	0.757	0.507 **	0.687 **	**0.714**	0.638 **
BI	3.71	0.69	0.923	0.909	0.638 **	0.643 **	0.638 **	**0.845**

**^a^** CR = composite reliability; ** *p* ≤ 0.01; figures in boldface denote the square root of average value extract (AVE); AT = attitude; SN = subjective norms; PBC = perceived behavioural control; PBE = perceived benefit of vitamin D; BI = supplementation intention.

**Table 4:** Effects of ANC on BI and perceived benefit.

Associations	Estimate	S.E.	C.R.	*p*	Std.	
BI←-AT	0.286	0.071	3.999	***	0.261	H1 was supported
BI←-PBC	0.236	0.106	2.241	0.025	0.183	
BI←-SN	0.168	0.080	2.095	0.036	0.169	
BI←-PBE	0.322	0.067	4.806	***	0.310	
PBE←-AT	0.290	0.073	3.951	***	0.275	H3 was supported
PBE←-PBC	0.369	0.109	3.368	***	0.296	
PBE←-SN	0.168	0.086	2.584	0.010	0.230	

BI = vitD supplementation intention; PBE = perceived benefit of vitD; AT = attitude; PBC = perceived behavioural control; SN = subjective norm; S.E. = standard error; C.R. = critical ratio; *** *p* < 0.001.

**Table 5:** Standardized effects of ANC and PBE on BI (BCPM).

	Total Effects			Direct Effects			Indirect Effects		
Confidence I.	CI1	CI2	*p*	CI1	CI2	*p*	CI1	CI2	*p*
BI←-AT	0.193	0.489	0.002	0.101	0.431	0.005	0.030	0.176	0.002
BI←-SN	0.052	0.440	0.029	0.012	0.365	0.042	0.015	0.156	0.017
BI←-PBC	0.058	0.484	0.018	0.011	0.376	0.050	0.037	0.179	0.002
BI←-PBE	0.163	0.473	0.004	0.163	0.473	0.004			

BCPM = bias-corrected percentile methods; CI1 = confidence interval, lower bound; CI2 = confidence interval, upper bound; AT = attitude; SN = subjective norms; PBC = perceived behavioural control; PBE = perceived benefit of vitamin D; BI = supplementation intention.

## Data Availability

The data supporting the reported results will be available upon request.

## Supplementary Materials

List of items in the questionnaire can be found at https://www.mdpi.com/article/10.3390/ijerph19041952/s1; other research materials are available upon request.
