# Supplementary material for: Effects of Perceived Benefit on Vitamin D Supplementation Intention: A Theory of Planned Behaviour Perspective
Source: Int J Environ Res Public Health. 2022 Feb 10;19(4):1952. doi: 10.3390/ijerph19041952 (PMC8872502; doi:10.3390/ijerph19041952)
Supplement: Supplementary file 1 [file ijerph-19-01952-s001.zip › ijerph-1424349-supplementary.pdf]

Items for TPB variables (after pilot test)

Code	Description
TA1	Regularly taking vitamin D supplement is helpful to body health
TA2	Regularly taking vitamin D supplement is valuable to body health
TA3	Regularly taking vitamin D supplement is good to maintain good health
TA4	Regularly taking vitamin D supplement is a pleasant expereince for body health
TC1	Regularly taking vitamin D supplement is possible
TC2	If I wanted I could take vitamine D each day in the forthcoming month
TC3	Regularly taking vitamin D supplement is completely out of my own control
TS1	My family members who live with me take vitamin D supplement each day for body health
TS2	My colleagues of current job take vitamin D supplement each day for body health
TS3	My friends since childhood take vitamin D supplement for body health
TS4	My friends in the clubs I belonged take vitamin D supplement to maintain their body health
TS5	My friends in the healthcarel industry also take vitamin D supplement to maintain their health
TI1	I will take vitamin D supplement each day for the coming month
TI2	Even if the cost of take vitamin D supplement becomes higher, I will continue to use it each day
TI3	I plan to consume vitamin D supplement each day for the coming month
TI4	Even if I am out of town for a journey, I will try to take vitamin D supplement each day
